# Scaling up area-based conservation to implement the Global Biodiversity Framework’s 30x30 target: The role of Nature’s Strongholds

**DOI:** 10.1371/journal.pbio.3002613

**Published:** 2024-05-21

**Authors:** John G. Robinson, Danielle LaBruna, Tim O’Brien, Peter J. Clyne, Nigel Dudley, Sandy J. Andelman, Elizabeth L. Bennett, Avecita Chicchon, Carlos Durigan, Hedley Grantham, Margaret Kinnaird, Sue Lieberman, Fiona Maisels, Adriana Moreira, Madhu Rao, Emma Stokes, Joe Walston, James EM Watson

**Affiliations:** 1 Wildlife Conservation Society, Bronx, New York, United States of America; 2 34 Kibo Lane, Karen, Kenya; 3 Equilibrium Research, Bristol, United Kingdom; 4 Andes-Amazon Initiative, Gordon and Betty Moore Foundation, Palo Alto, California, United States of America; 5 Wildlife Conservation Society Brasil, Manaus, Amazonas, Brazil; 6 Center for Ecosystem Science, School of Biological, Earth and Environmental Sciences, University of New South Wales, Sydney, New South Wales, Australia; 7 Bush Heritage Australia, Melbourne, Victoria, Australia; 8 World Wide Fund for Nature International, Gland, Switzerland; 9 Wildlife Conservation Society Congo, Brazzaville, Republic of Congo; 10 Biological and Environmental Sciences, University of Stirling, Stirling, United Kingdom; 11 Global Environmental Facility, Washington, DC, United States of America; 12 World Commission on Protected Areas, International Union for Conservation of Nature, Gland, Switzerland; 13 School of The Environment, University of Queensland, Brisbane, Queensland, Australia

## Abstract

The Global Biodiversity Framework (GBF), signed in 2022 by Parties to the Convention on Biological Diversity, recognized the importance of area-based conservation, and its goals and targets specify the characteristics of protected and conserved areas (PCAs) that disproportionately contribute to biodiversity conservation. To achieve the GBF’s target of conserving a global area of 30% by 2030, this Essay argues for recognizing these characteristics and scaling them up through the conservation of areas that are: extensive (typically larger than 5,000 km^2^); have interconnected PCAs (either physically or as part of a jurisdictional network, and frequently embedded in larger conservation landscapes); have high ecological integrity; and are effectively managed and equitably governed. These areas are presented as “Nature’s Strongholds,” illustrated by examples from the Congo and Amazon basins. Conserving Nature’s Strongholds offers an approach to scale up initiatives to address global threats to biodiversity.

## Abstract 2

The 2022 Global Biodiversity Framework set out target of conserving a global area of 30% by 2030. This Essay provides a framework for area-based conservation that preferences "Nature’s Strongholds", arguing that these areas are disproportionately important for the conservation of biodiversity.

## Introduction

The Kunming–Montreal Global Biodiversity Framework (GBF), adopted at the 15th meeting of the Conference of Parties to the UN Convention on Biological Diversity (CBD) in Montreal [1], recognized the importance of area-based conservation to deliver on the overarching biodiversity goal of the GBF (Goal A): “The integrity, connectivity, and resilience of all ecosystems are maintained, enhanced, or restored, substantially increasing the area of natural ecosystems by 2050.” Area-based conservation refers collectively to the use of both “protected areas,” as recognized by the International Union for the Conservation of Nature (IUCN) and CBD, and “other effective area-based conservation measures” (OECMs) [2].

The goal of protecting at least 30% of global land and ocean by 2030 (the 30x30 target) emerged from scientific studies that argue that greater area-based ambition is a necessary component of conservation policies if the loss of biodiversity is to be halted [3–6], and has been promoted by advocacy campaigns [7,8]. GBF Target 3 (Box 1) ambitiously builds upon and extends Aichi Target 11 [2], which specified a goal of protecting “at least 17 per cent of terrestrial and 10 per cent of coastal and marine areas,” made up of “protected areas and other effective area-based conservation measures.” Under the Aichi targets, there was significant growth in the area under protection but more limited gains in biodiversity protection [9].

**Box 1. Characteristics of protected and conserved areas identified in the Global Biodiversity Framework as important for biodiversity conservation**

To deliver on its biodiversity goal, the Global Biodiversity Framework (GBF) has explicit targets.

- GBF Target 1 addresses the need for effective planning and management and equitable governance. It seeks to “ensure that all areas are under participatory, integrated and biodiversity inclusive spatial planning and/or effective management processes addressing land- and sea-use change, to bring the loss of areas of high biodiversity importance, including ecosystems of high ecological integrity, close to zero by 2030, while respecting the rights of indigenous peoples and local communities.” It also prioritizes the ecological integrity of conservation areas.
- GBF Target 2 seeks to “ensure that by 2030 at least 30% of areas of degraded terrestrial, inland water, and coastal marine ecosystems are under effective restoration, in order to enhance biodiversity and ecosystem functions and services, ecological integrity and connectivity.” This target, among other considerations, notes that connectivity and ecological integrity are integral to area-based conservation.
- GBF Target 3 formally links a strategy for area-based conservation to the biodiversity outcomes of the numerical target of conserving 30% of the globe by 2030. Protected and conserved areas need to be effectively managed and equitably governed, interconnected, and embedded in larger conservation landscapes. Protected and conserved areas explicitly include both traditional protected areas and “other effective area-based conservation measures.” The target is to “ensure and enable that by 2030 at least 30 per cent of terrestrial, inland water, and of coastal and marine areas, especially areas of particular importance for biodiversity and ecosystem functions and services, are effectively conserved and managed through ecologically representative, well-connected and equitably governed systems of protected areas and other effective area-based conservation measures… and integrated into wider landscapes and seascapes…”.

A consensus around conserving 30% by 2030 gained political momentum leading up to the UN CBD Biodiversity Conference (COP15) in November 2022. On assuming office in 2021, President Biden issued an Executive Order that committed the United States to the goal of conserving 30% of its lands and waters by 2030 [10]. In June 2021, the G7 members committed in their Nature Compact “to conserve or protect at least 30% of global land and at least 30% of the global ocean by 2030.” In the build up to COP15, over 100 countries joined the High Ambition Coalition to champion the 30x30 target [11], and over 70 countries joined the Global Ocean Alliance [12]. This enthusiasm has also translated into increased funding: In addition to commitments made at COP15, the “Protecting our Planet Challenge” was launched at the UN Climate Change Conference (COP26) in Glasgow and represents a $5 billion commitment to support the protection of at least 30% of the planet in the most important areas for biodiversity by 2030 [13]; and in June 2023, the Council of the Global Environmental Facility approved plans to establish the Global Biodiversity Fund to support implementation of the GBF. The efficacy for biodiversity conservation of the 30x30 target depends on where protected areas are located, and how they are configured and managed [14].

The GBF has specified the characteristics of protected and conserved areas (PCAs) that are important for biodiversity conservation (Box 1). In this Essay, we consider these characteristics and identify 4 criteria that we argue should be prioritized and scaled up in order to strengthen biodiversity outcomes: PCAs should be extensive; interconnected (either physically or as part of a jurisdictional network, and frequently embedded in larger landscapes); have high ecological integrity; and be effectively managed and equitably governed. We suggest that specific areas that incorporate all 4 criteria, areas that we call “Nature’s Strongholds,” are disproportionately important for the conservation of biodiversity and need to be prioritized for safeguarding if the mission of the GBF is to be achieved. Using these criteria, we look at how to identify such strongholds, providing examples in the river basins of Central Africa and the Amazon. These regions are both high-biodiversity tropical forest regions with a tradition of area-based conservation, but they exhibit variation in the size of single or mosaics of PCAs, the extent of the conservation landscape in which strongholds are embedded, the pattern of ecological integrity across the area, and PCA management and governance regimes.

## Characteristics contributing to area-based conservation of biodiversity

### Size of PCAs

The species-area curve, a fundamental ecological relationship, describes that as the size of an area increases, the extent of natural habitat and the number of species present also increase. Conversely, biodiversity can be lost simply as the area of natural habitat is diminished [15], or through the differential loss of ecosystems and their associated species and biological communities [5,16]. More generally, the loss of large, contiguous natural areas drives biodiversity loss [17–20].

Conservation areas retain natural habitat, but creating large PCAs does not by itself produce biodiversity outcomes: they need to be located in the right places [21]. Conservation areas should be located in geographic areas that contain abundant biodiversity and connected to similar areas. Nevertheless, increasing the size and compactness of single PCAs or mosaics of PCAs decreases the proportion of natural habitat located close to areas that are unprotected or have other land uses, making them less vulnerable to many anthropogenic stressors, including climate change [22]. Large PCAs can also contain many natural habitats and the ecological and evolutionary processes that sustain them.

### Connectivity of areas

The terms “connectivity” and “well-connected systems,” as referenced in the GBF, we interpret as referring to both the physical or ecological connectivity of natural areas (such as PCAs that are physically contiguous or linked through corridors) and to management connectivity (such as multiple PCAs that might not be physically contiguous but are linked jurisdictionally and embedded in a larger natural landscape matrix, providing for ecological connectivity and management across jurisdictional boundaries). Connectivity, as a result of either condition, allows the movement of species across the landscape and seascape, increasing effective population sizes, and allows animals, especially those with behavioral flexibility, to have access to suitable environmental conditions in the context of climate change and sufficient resources even in times of ecological stress [23–25]. Conversely, the fragmentation of natural habitats and the loss of connectivity across a landscape is strongly associated with the loss of biodiversity [26,27]. Fragmentation changes ecological processes, and smaller habitat fragments have less biodiversity than would be expected from the loss of habitat alone [28].

Aggregations or mosaics of PCAs under multiple jurisdictions would allow the alignment of conservation goals across larger areas. Defining what categories of PCAs should be included in these mosaics so as to meet the GBF 30x30 target [29] remains a work in progress. There is a good consensus that all 6 IUCN categories of protected areas (Ia, strict nature reserve; Ib, wilderness area; II, national park; III, natural monument; IV, habitat or species management area; V, protected landscape or seascape; VI, protected areas with sustainable use of natural resources) should be included, although they are not simply interchangeable. Furthermore, they should demonstrably deliver on biodiversity outcomes [30]. Because effectiveness of protected areas is typically measured by their attaining management objectives rather than achieving biodiversity conservation [31], this is not always the case [32–35].

OECMs might also be included and contribute to the 30x30 target [14,36]. OECMs, in contrast to traditional protected areas, are governed by many different authorities, from national governments to private entities and civil society, to indigenous peoples and local communities, and might include indigenous and traditional territories (ITTs). Ongoing work by the IUCN, and specifically its World Commission on Protected Areas, seeks to establish a recognized method to define types of OECMs [37] and assess their possible contribution to reaching the 30x30 target.

Including the category of OECMs in the 30x30 target would enable countries to more easily attain biodiversity conservation goals. OECMs, by definition (CBD Decision COP XIV/8, 2018) explicitly contribute to biodiversity conservation [38]: An OECM is “a geographically defined area other than a Protected Area, which is governed and managed in ways that achieve positive and sustained long-term outcomes for the in situ conservation of biodiversity, with associated ecosystem functions and services and where applicable, cultural, spiritual, socioeconomic, and other locally relevant values.” In their delivery of biodiversity outcomes, OECMs can be favorably compared to formally protected areas. A series of detailed studies in Amazonia have compared deforestation and degradation rates among different categories of conserved and managed areas. ITTs compared favorably with areas under national jurisdiction and those supporting extractive activities [39–42]. A similar pattern is evident globally [43,44]. In addition, these areas have an extensive geographic distribution. ITTs, in particular, cover a large proportion of the planet and overlap with 40% of protected areas [45]. They overlap extensive areas of intact forest landscapes [46] and the ranges of many species, including those that are endangered [47].

While many existing areas under the jurisdiction of indigenous peoples and local communities have the potential to be recognized as OECMs, including such OECMs in the 30x30 tally will require that local customs are followed and/or will need to be approved by the relevant Indigenous peoples and local community actors through processes that respect human rights obligations [48], including free prior and informed consent and equitable benefit sharing and governance.

### Degree of ecological integrity

The concept of ecological integrity became a part of ecology’s lexicon with Aldo Leopold’s comment: “A thing is right when it tends to preserve the integrity, stability, and beauty of the biotic community. It is wrong when it tends otherwise” [49]. While there are different approaches to defining ecological integrity, there is general agreement that it can be characterized by the structure, composition, and function of natural ecosystems [50,51]. Efforts to quantitatively describe and measure ecological integrity have depended on measuring some characteristic of ecosystems that can be used as a proxy for integrity, be it structural (such as forest extent, the degree of fragmentation, or the size and frequency distribution of live trees), compositional (such as species occurrence and community composition), or functional (such as net primary productivity or energy and nutrient cycling) [52]. An alternative approach is quantifying measures of human pressure or modification of natural systems that are considered to systematically influence integrity (e.g., population density, land-use change, roads, extractive industries, light pollution) [53,54]. The first approach has been most useful at local and regional scales, where such direct measurement is more feasible. The second has had utility at more global scales, where proxies for pressure are available [17,20].

In response to the needs of the GBF for measures of integrity to study, manage, and report on biodiversity change, a hybrid approach to generate integrity indicators has gained traction. It combines measures of human pressure or modification with modeled measures of ecosystem properties, often based on remote sensing and/or direct observations [55]. For example, the Forest Landscape Integrity Index was developed on the basis of observed human pressure at a landscape level and then used to model loss of forest connectivity [56]. Similarly, the Contextual Intactness Index (CII) used the Human Footprint to infer a biodiversity value based on geographically explicit species occurrence from museum collections [33]. Methods have also been developed that combine measures of human pressure with empirical measurements of biodiversity across multiple scales [57]. For the GBF, the most useful indicators of ecological integrity should have a global application and a temporal resolution that enables periodic monitoring [58].

Within a given ecosystem, ecological integrity is a good predictor of high biodiversity [59] and is clearly important for climate adaptation [60,61]. Conversely, loss of habitat and connectivity results in loss of ecological integrity, which erodes biodiversity [62]. The major driver of these patterns is that the loss of ecological integrity increases the probabilities of local extinction [19]. For example, high-integrity tropical rainforests, as measured by structural intactness, are associated with lower risks of species extinction for tropical mammals, birds, reptiles, and amphibians across all biogeographic realms [27], and ecological integrity of Southeast Asian tropical forests can be used to predict actual extirpations of megafauna during the Holocene and/or Anthropocene [63].

### PCA management and governance

While the GBF does not define when an area is “effectively managed,” traditionally the term has been interpreted as reflecting the extent to which the goals and objectives for the area are achieved [64]. Many studies have examined the constraints of effective management for conservation areas (e.g., the need for adequate funding [65], capacity shortfalls [66], and adequate personnel [67]). A widely used self-reporting tool to monitor protected areas is the Management Effectiveness Tracking Tool (METT) [68].

While many studies have demonstrated that protected areas are important for biodiversity conservation [69], few studies have directly measured the importance of management effectiveness per se. One such study reported that species populations in protected areas were positively associated with the area’s METT score [70]. The authors then went on to argue that “documenting the delivery of biodiversity outcomes must be an explicit part of any future assessment of effectiveness” [71].

The GBF also urges that areas be “equitably governed.” While scientific assessments of the extent to which and how protected areas meet this requirement are still largely lacking, CBD Decision COP XIV/8, Annex II (2018) provides guidance on how this might be measured: appropriate procedures should be in place to ensure that the diversity of “rights holders” and stakeholders are recognized, that rule making and decision-making are inclusive, and the costs and benefits are equitably shared. Effective governance requires that “duty bearers” provide timely and competent assistance to rights holders. Dudley and colleagues argue that conserved and managed areas should only be recognized as contributing to the 30x30 target when authorities or duty bearers recognize and respect rights holders and stakeholders, and provide the ecosystem services to meet human needs [30]. Tools are becoming available for measuring the effectiveness of governance and social outcomes in PCAs [72–75]. The expectation is that when this is the case, the “conserved and managed” areas of GBF Target 3 will have greater permanence through political and legal support, greater stakeholder buy-in, and access to more financial and other resources. There is some supporting data for this expectation [76].

## Identifying Nature’s Strongholds

How these characteristics affect biodiversity conservation will vary geographically and ecologically. The identification of Nature’s Strongholds will be affected, in a specific region, by the size and distribution of PCAs, the continuity or fragmentation of the natural matrix, the spatial pattern of ecological integrity, and the existing governance and management regimes. In considering their application to the Central African and Amazonian river basins, our 2 case studies, we interpreted the characteristics as follows, and defined explicit criteria that were appropriate for these 2 regions.

1. **Large protected and conserved areas.** Our assumption was that PCAs needed to be large enough to maintain biodiversity. There is no consensus around the desired size of PCAs, but a range of sizes have been proposed. To protect functioning ecosystems, the IUCN established a global Standard for Key Biodiversity Areas [77] and suggested a possible size threshold of 10,000 km^2^. More of a focus on tropical regions has defined the work of some other organizations: the Wildlife Conservation Society (WCS) has structured its area-based work around areas with a minimum size of 5,000 km^2^ [78]; the German Government’s “Legacy Landscapes” program [79] suggests a minimum of 2,000 km^2^; and African Parks, a non-governmental organization (NGO) focused on park management, identified “core anchor areas” in Africa of disproportionate importance for biodiversity conservation, with a minimum size of 500 km^2^. For the case studies, we gave preference to larger areas, and arbitrarily identified PCAs (single or as aggregations) that were approximately 5,000 km^2^ or larger.
2. **Interconnected areas.** In addition to identifying strongholds where a PCA was sufficiently large, we looked for groups of PCAs that were physically or ecologically contiguous, thus creating a larger conservation area, and groups of PCAs that, although not physically contiguous, were embedded in the same conservation landscape, often with jurisdictional commonalities and management coordination across the landscape. In both Central Africa and Amazonia, the definition and identification of strongholds was aided by their being embedded within larger “Key Landscapes for Conservation” (KLCs), which had previously been identified in studies supported by the European Union (EU) [80,81]. In addition, to inform their philanthropy, the Gordon and Betty Moore Foundation (GBMF) has identified a suite of conservation landscapes in Amazonia [82] that generally align with the EU analysis. The resulting landscapes that contained identified strongholds were large. In Central Africa, the average size was 62,257 km^2^ (*n* = 16) and in Amazonia, the average size was 217,488 km^2^ (*n* = 14).
3. **Effectively managed and equitably governed PCAs.** Systems of governance and management vary in different parts of the world, and the criteria associated with PCAs will always be politically and culturally specific. In Central Africa, national government agencies typically retain authority over most PCAs (including national parks, nature reserves, faunal reserves, and wildlife reserves), although devolved authority characterizes community reserves, forest management units, and local community forest concessions. In addition, collaborative management partnerships through an agreement between government and international or national NGOs are increasingly common. In Amazonia, in addition to PCAs managed by national governments (e.g., national parks, wildlife reserves), PCAs managed by states and municipalities are common. Devolved authority to a local level characterizes extractive reserves, ITTs, and sustainable development reserves. Further management and governance criteria used to identify strongholds are described below separately for Central Africa and Amazonia.
4. **High ecological integrity.** We used Mokany and colleagues’ [33] CII to measure ecological integrity. The index infers a biodiversity value and uses the Human Footprint [53] to assess human impact. We did not use ecological integrity in the initial identification of strongholds, as we had no a priori rationale for defining a minimum level of integrity for strongholds. Nevertheless, we expected that ecological integrity would covary with the other characteristics. Therefore, once strongholds were identified using the first 3 criteria, we compared their integrity to that of the conservation landscapes in which they were embedded and compared the integrity of the landscapes to the river basin as whole. A possible stronghold where its ecological integrity was lower than the landscape in which it was embedded was not included.

## Case study 1: Nature’s Strongholds in Central Africa

Identification of Nature’s Strongholds in Central Africa initially depended on 3 of the criteria: large single or mosaics of PCAs of approximately 5,000 km^2^ or larger, embedded within previously defined conservation landscapes, with demonstrably effective management and a commitment to equitable governance. The process was also informed by previous analyses of priority areas and management effectiveness [80,83,84], any international recognition (such as by a World Heritage Site designation), and expert opinion from managers of PCAs.

To delineate strongholds, we were helped by reference to the EU conservation strategy for Africa “Larger than Elephants” [80], which identified KLCs, including 20 in Central Africa. KLCs bounded individual strongholds, which could be single or multiple jurisdictional distinct PCAs within a single KLC (some large KLCs that crossed national boundaries were subdivided; see S1 Text). Table 1 lists the KLCs which contained identified strongholds (see S1 Text). Identified strongholds frequently included multiple PCAs.

**Table 1:** KLCs and Nature’s Strongholds in Central Africa.

KLC^a^	KLC area (km^2^)	KLC mean CII	Stronghold	PCAs	PCA area (km^2^)	PCA mean CII	PCA categories^b^	Institutional context^c^
CAF 01 (Cross River–Takamanda–Mt. Cameroon–Korup)	27,418	0.38	Cross River–Takamanda Complex	Cross River	3,297	0.54	NP (IUCN II)	WCS
				Takamanda	628	0.61	NP (IUCN II)	WCS
				Mt. Cameroon	582	0.32	NP (IUCN II)	None
CAF 03 (Greater Tri-National); 3a. Cameroon	54,476	0.69	Tri-National Cameroon	Dja	5,265	0.82	FR (IUCN IV)	WHS
CAF 03 (Greater Tri-National); 3b. Gabon	76,146	0.75	Tri-National Gabon	Lopé	4,945	0.75	NP	None
				Ivindo	2,969	0.82	NP	None
				Minkebe	7,539	0.91	NP	WWF
CAF 03 (Greater Tri-National); 3c. Republic of Congo	48,444	0.83	Tri-National Congo	Odzala Kokoua	13,604	0.88	NP (IUCN II)	AP^d^
CAF 03 (Greater Tri-National); 3d. Sangha Tri-National (Cameroon, Republic of Congo and Central African Republic)	77,099	0.79	Sangha Tri-National	Dzanga-Sangha	3,393	0.82	NP (IUCN IV)	WHS, WWF^d^
				Nouabalé-Ndoki	4,089	0.92	NP (IUCN II), UFA	WCS^d^
				Lobéké	2,155	0.81	NP (IUCN II)	WWF
				Lac Télé	4,511	0.87	CR (IUCN IV)	WCS
				Ntokou-Pikounda	4,254	0.84	NP	WWF^d^
CAF 04 (Gamba–Mayumba–Conkouati)	72,945	0.49	Gamba—Conkouati	Gamba Complex (Loango, Iguela, Moukalaba doudou)	12,308	0.76	NP (IUCN II,IV)	None
				Mayumba	17	0.38	NP	None
				Conkouati	5,134	0.70	NP (IUCN II)	PN^d^
CAF 05 (Garamaba-Bili Uere–Chinko–Zemongo–Southern); 5a. Central African Republic	103,398	0.91	Chinko	Chinko	24,646	0.98	NP	AP^d^
CAF 05 (Garamaba-Bili Uere–Chinko–Zemongo–Southern); 5b. Democratic Republic of Congo	99,640	0.69	Garamba	Garamba	4,949	0.81	NP (IUCN II)	WHS, AP^d^
CAF 05 (Garamaba-Bili Uere–Chinko–Zemongo–Southern); 5c. South Sudan	95,581	0.60	Southern	Southern	19,239	0.65	NP (IUCN II)	None
CAF 06 (Gounda–St. Floris–Bamingui and surrounding hunting blocks)	123,246	0.90	Northeastern Protected Area Complex, Central Africa Republic	Manovo–Gounda–St. Floris	20,082	0.94	NP (IUCN II, IV)	WHS, WCS^d^
				Bamingui-Bangoran	11,191	0.98	NP (IUCN II)	WCS^d^
CAF 07 (Salonga)	66,625	0.75	Salonga	Salonga	33,368	0.81	NP (IUCN II)	WHS, WWF
CAF 08 (Okapi)	37,614	0.79	Okapi	Okapi	13,940	0.70	WR (IUCN IV)	WHS, WCS^d^
CAF 09 (Kahuzi-Biega)	18,181	0.67	Kahuzi-Biega	Kahuzi-Biega	6,731	0.57	NP (IUCN II), CFCL	WHS, WCS^d^
CAF 10 (Maiko-Tayna)	30,092	0.65	Maiko	Maiko	10,968	0.78	NP (IUCN II)	None
CAF 14a (Itombwe)	10,442	0.17	Itombwe—Kabobo	Itombwe	6,033	0.44	NR	None
CAF 14b (Kabobo)	6,784	0.50		Kabobo	1,870	0.53	WR, CFCL	WCS
CAF 15 (Lomami)	30,925	0.78	Lomami	Lomami	8,875	0.80	NP	FZS
CAF 16 (Mbam and Djerem)	17,499	0.70	Mbam Djerem	Mbam and Djerem	4,228	0.78	NP (IUCN II)	WCS
				Deng Deng	687	0.64	NP (IUCN II), UFA	WCS
CAF 18 (Zakouma–Sinlaka Minla)	47,710	0.58	Zakouma Complex	Zakouma	3,043	0.63	NP (IUCN II)	AP^d^
				Siniaka–Minla	4,307	0.75	WR (IUCN IV)	AP^d^
				Bahr Salamat	21,208	0.59	WR (IUCN IV)	AP^d^

^a^CAF codes follow [80].

^b^PCA categories are listed in this column: CFCL, local community forest concession; CR, community reserve; FR, faunal reserve; NP, national park; NR, nature reserve; UFA, forest management unit; WR, wildlife reserve. Also listed are IUCN Protected Area Categories: Category II = national park; Category IV = habitat or species management area (not all PCAs in a country follow IUCN categories, or are recognized presently by the IUCN).

^c^Partnerships in PCAs with non-governmental organizations are listed in this column: AP, African Parks; FZS, Frankfurt Zoological Society; IUCN, International Union for Conservation of Nature; PN, Parc Noé; WCS, Wildlife Conservation Society; WWF, World Wide Fund for Nature. Protected areas designated as World Heritage Sites (WHS) are noted.

^d^These are Collaborative Management Partnerships through a formal agreement between the government and a non-governmental organization.

CAF, Central Africa codes for KLCs; CII, Contextual Intactness Index; KLC, Key Landscape for Conservation; PCA, protected and conserved area.

**Fig 1:**
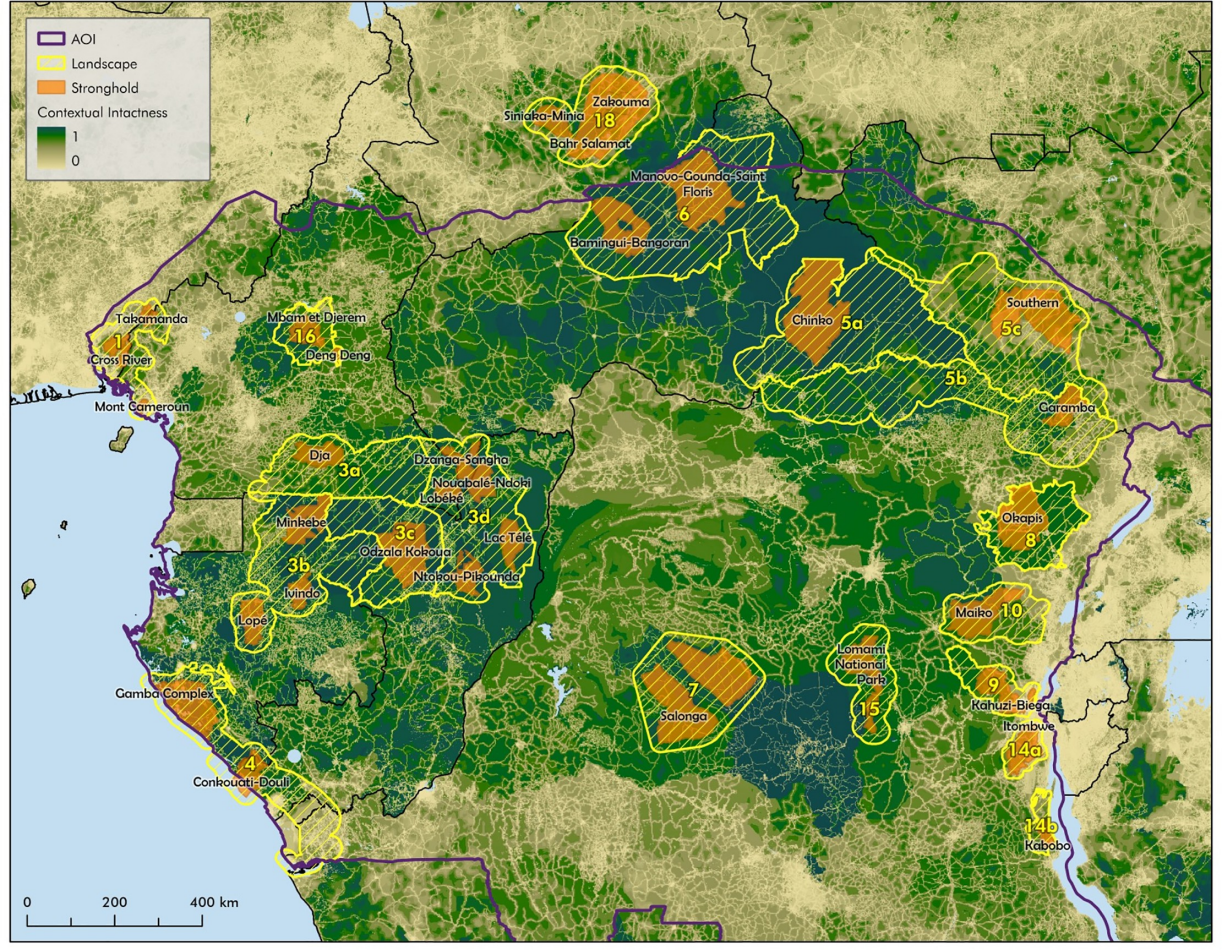
Nature’s Strongholds in Central Africa. KLCs and Nature’s Strongholds in Central Africa (EU identified KLCs numbered, embedded protected and conserved areas constitute the identified strongholds) mapped onto ecological integrity of the region, as measured by the CII. Data layers used are listed in S1 Text. AOI, area of interest; CII, Contextual Intactness Index; EU, European Union; KLC, Key Landscapes for Conservation.

Within a KLC, PCAs were grouped into strongholds if their physical or jurisdictional configuration, management, funding, or institutional context were aligned. In some cases (e.g., Odzala Kakoua, CAF 03c in Fig 1), individual PCAs were sufficiently large to define a stronghold, in others (e.g., Gamba complex, CAF 04 in Fig 1), individual PCAs were contiguous forming a mosaic, and in still others (e.g., PCAs in CAF 03d in Fig 1), where PCAs were not contiguous, they occurred in a single conservation landscape with jurisdictional links and management across the landscape. One alignment, and an indication of more effective management, is if there is a significant management partnership between national governments and international NGOs (see Table 1), with the concomitant international donor funding that comes with these relationships. Africa has been in the forefront of defining collaborative management models [83]. Conservation management partnerships offer a range of governance mechanisms between governments, local communities, private entities, and NGOs, sometimes involving joint ventures and delegated management authority [85,86].

Comparisons of CII values of strongholds and their surrounding KLCs were used to confirm the identification of individual strongholds. In all cases, with 1 exception, the ecological integrity of identified strongholds was greater than the KLCs in which they were embedded. In the exception, the mean CII of Bouba Ndjida-Benoue KLC (CAF 17) was 0.73, while the mean CII values for the 2 PCAs in the stronghold, Bouba Ndjida in northern Cameroon and Sena Oura in Chad, were lower (0.68 and 0.42, respectively); this potential stronghold was therefore not included. In 2 other cases (Mt. Cameroon in CAF 01 and Mayumba in CAF 04), CII values of individual PCAs were lower than for the KLC, but the stronghold as a whole was higher, so the strongholds were retained.

In total, we identified 18 strongholds in Central Africa (average size = 15,003 km^2^). Each was located within a KLC and included one or more PCAs, often not physically contiguous. Possible strongholds were excluded if: PCAs, either singly or as aggregations, were much smaller than 5,000 km^2^; they fell outside of KLCs; there was little evidence of effective management or good governance; and the stronghold had a lower ecological integrity than the surrounding KLC. Identified strongholds and their surrounding KLCs were mapped onto the geographic distribution of ecological integrity across the basin, using the CII [33,87] (Fig 1). Identifying strongholds is a work in progress, and currently excluded areas could meet the criteria in the future through ecological restoration, provision of adequate funding and staffing, and strengthening management and good governance.

**Fig 2:**
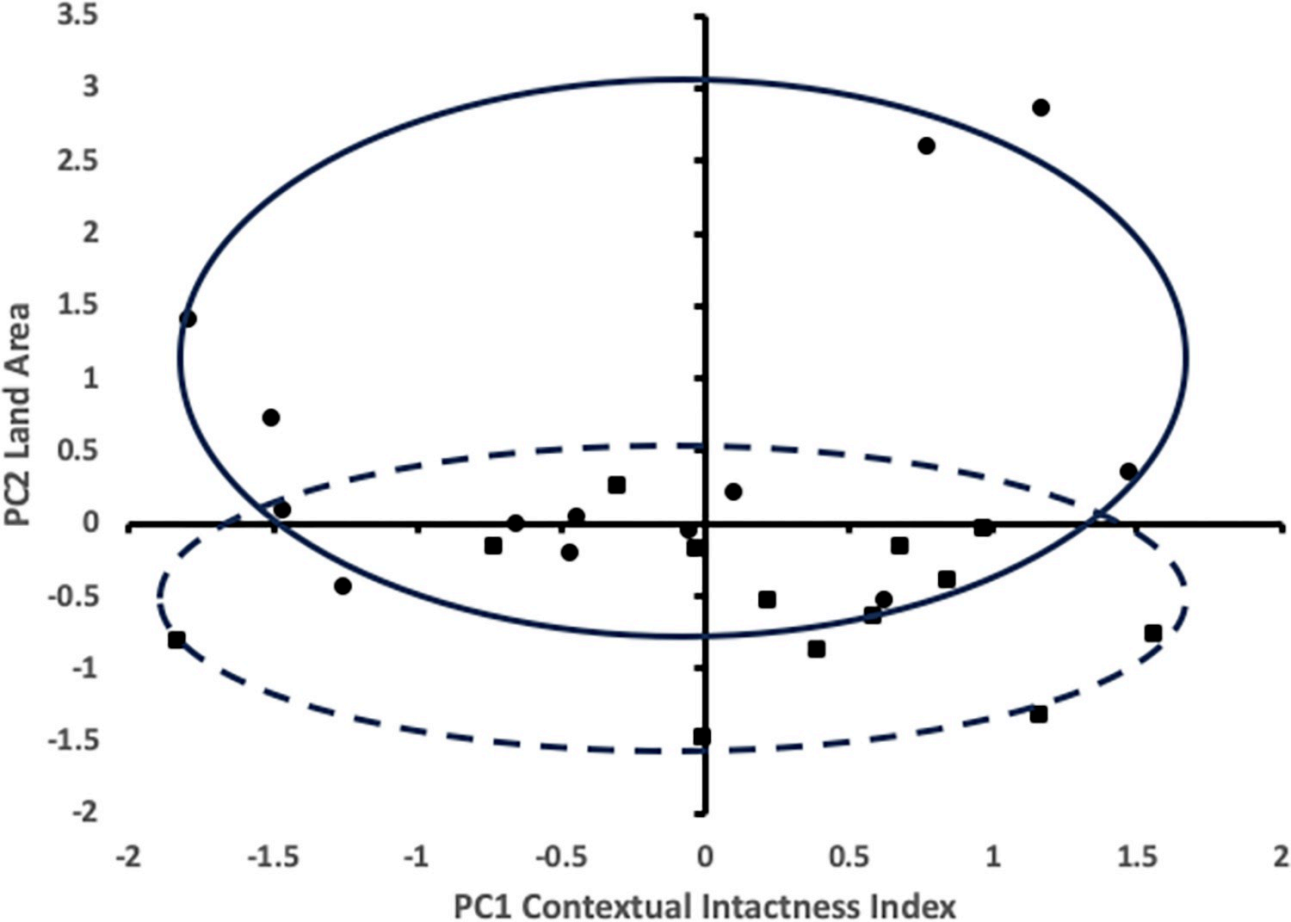
Ecological integrity of Nature’s Strongholds in Central Africa. Principal component analysis for Strongholds (filled squares, dashed oval) and Central African KLCs (filled circles, solid oval). Principal component 1 (PC1), which is most heavily weighted towards the CII, is plotted against principal component 2 (PC2), which is most heavily weighted towards land area. The ovals highlight the distribution of points in the stronghold class and the KLC class. They serve to easily show the degree of separation and are not a statistical representation. CII, Contextual Intactness Index; KLC, Key Landscapes for Conservation.

To make the overall case that strongholds (or their constituent PCAs) are more ecologically intact than the KLCs in which they are embedded (excluding the area of the stronghold itself), we used principal component analysis to compare, for all 1 km grid cells, the CII, the standard deviation of CII, and the land area with values scaled to have a mean of 0 and a variance of 1 (see Fig 2, S1 Text, and S1 Table). The first component is most heavily weighted toward the CII itself (0.893), followed by decreasing standard deviation of CII (−0.654), and the size of KLCs and strongholds (0.406). This means that higher values of ecological integrity are associated with larger areas and lower variance of ecological integrity. The second component is primarily weighted by size of the area (0.846), followed by increasing standard deviation of CII (0.650), and least by the CII itself (0.091). This means that larger areas have a higher variance in ecological integrity, but are no more intact than smaller areas. Each axis accounts for similar levels of variance in the data: 46.3% for principal component 1 and 38.2% for principal component 2, for a total of 84.5% of the total variance. Ecological integrity is higher in larger KLCs than smaller, and less variable in larger KLCs (Fig 2). By contrast, larger strongholds are not more ecologically intact than smaller ones. These observations were confirmed using paired *t* tests, which indicates that strongholds have both a higher average ecological integrity and a lower variance in ecological integrity than the KLCs in which they were embedded (see S1 Text).

We also looked at whether KLCs that contained identified strongholds were more ecologically intact than the Congo Basin as a whole (see S1 Text and S2 Table), and concluded that strongholds were of higher ecological integrity than the KLCs in which they are embedded, and combined KLCs (including embedded strongholds) are of higher ecological integrity than the Congo Basin as a whole.

To demonstrate that the distribution of large-bodied mammals (another proxy for biodiversity) maps onto Nature’s Strongholds, we examined the geographic distribution of forest elephants and great apes in Central Africa (S3 Table). The forest elephant (*Loxodonta cyclotis*) population was last estimated [88] at 24,119 ± 2,865, with an additional 87,190 to 103,355 in areas not systematically surveyed. Nine of the 32 identified strongholds contained populations numbering in the thousands, and 8 more had populations numbering in the hundreds. Similarly, Great Ape populations are found in strongholds [89]. Over 95% of the world’s remaining Cross River gorillas are found in Cross River and Takamanda parks, which also contain a population of the Nigeria-Cameroon chimpanzee (*Pan troglodytes elliotii*). The Western Lowland gorilla (*Gorilla gorilla gorilla*) and the Central chimpanzee (*Pan troglodytes troglodytes*) are found in strongholds and KLCs in Gabon, Cameroon, and the Republic of Congo; the Grauer’s gorilla (*Gorilla beringei graueri*) and the Eastern chimpanzee (*Pan troglodytes schweinfurthii*) are found especially in strongholds in the eastern Democratic Republic of Congo; and the bonobo (*Pan paniscus)* is largely restricted to the Democratic Republic of Congo protected areas of Lomami and Salonga.

## Case study 2: Nature’s Strongholds in Amazonia

Nature’s Strongholds in Amazonia were identified using similar criteria to those for Central Africa. To delineate strongholds, we referred to the EU “Larger than Jaguars” conservation strategy for Latin America [81], which defined KLCs for Amazonia, and to the GBMF-identified conservation landscapes in the Amazon basin [82].

Within these larger landscapes, strongholds were identified if PCAs were large or could be grouped into larger aggregations, were interconnected, and were effectively managed and governed. In Amazonia, in contrast to Central Africa, in all cases, aggregations of individual PCAs were always physically contiguous. PCAs included protected areas, ITTs, sustainable development reserves, extractive reserves, and other conservation areas. Table 2 lists the KLCs and GBMF mosaics, which contain identified strongholds.

**Table 2:** KLCs, GBMF mosaics, and Nature’s Strongholds in Amazonia.

KLC^a^	GBMF conservation landscape	Stronghold^b^	Stronghold area (km^2^)	Stronghold mean CII	Surrounding landscape area (km^2^)	Surrounding landscape mean CII	PCA categories (within stronghold)^c^	Institutional context^d^
No KLC	Calha Norte	1 (Eastern Amazon)	124,355	0.78	279,924	0.57	NP	ARPA, MOS
43 (Terra do Meio)	Xingu	2 (Xingu–Kayapo)	263,006	0.84	496,531	0.48	ES, IT, ER	ARPA
44 (Tapajós river basin)	Madeira	3 (Apui–Southern Amazon)	62,968	0.81	162,328	0.68	NP, SP, ER	ARPA, MOS
45 (Purús river basin)		4 (Purus–Madeira Interfluvial)	26,855	0.77	91,812	0.64	NP, SDR	ARPA
		5 (Mapinguari)	48,016	0.81	148,727	0.60	NP, ER	ARPA
45 (Negro river basin)	Lower Rio Negro	6 (Mamiráua–Amanã–Jaú-Unini)	66,199	0.78	161,309	0.69	NP, SDR, ER	ARPA, MOS
51 (Peru–Ecuador–Colombia border)	Yasuni-Pastaza	7 (Yasuní-Cuyabeno)	29,089	0.76	90,269	0.59	NP, WR	None
52 (Chiribiquete)	Chiribiquete–Caqueta	8 (Chiribiquete–Caqueta)	86,885	0.76	197,506	0.47	NP, IT	HECO
55 (Cordillera Azul–Pacaya Samiria)	Yavari–Samiria	9 (Pacaya Samiria)	21,718	0.78	77,315	0.65	NR	None
55 (Sierra del Divisor)		10 (Divisor)	22,782	0.80	90,248	0.69	NP	ARPA
56 (Javari)		11 (Javari)	85,331	0.77	171,354	0.65	IT	None
57 (Chico Mendes–Cazumbá–Iracema)	Upper Purus	Brazilian part of 12	Included below	Included below	Included below	Included below	SP, ER	ARPA
58 (Madre de Dios–Manu–Alto Purús)		12 (Manu–Alto Purús)	58,161	0.74	164,281	0.58	NP	PdP
59 (Madidi–Manuripi–Mojos)	Madidi–Tambopata	13 (Madidi)	63,373	0.85	149,160	0.43	NP, NAIM, BR, MPA, IT	None
60 (Iteñez river basin)	(Iteñez–Rondonia)	14 (Noel Kempff Mercado)	18,568	0.71	68,703	0.53	NP	None

^a^KLC numbers follow [81].

^b^PCAs within each stronghold are listed: **1**, Montanhas do Tucumaque NP; **2**, Terra do Meio ER, Kayapó IT, Menkragnoti IT, Xingu IT, Capoto/Jarina IT.,Araweté Igarapé Ipuxuna IT, Ituxi ER; **3**, Juruena NP, Campos Amazônicos NP, Guariba SP, Sucunduri SP, Guariba ER, Guariba-Roosevelt ER; **4**, Nascenentes do Lago Jari NP, Serra da Cutia NP, Piagaçu-Purus SDR; **5**, Mapinguari NP, Médio Juruá ER, Ituxi ER; **6**, Jaú NP, Mamiráua SDR, Amanã SDR, Rio Unini ER; **7**, Yasuní NP, Cuyabeno WR; **8**, Chiribiquete NP, Yaigoje-Apoporis NP, Miriti IT; **9**, Pacaya Samiria NR; **10**, Sierra Del Divisor NP, Serra do Divisor NP; **11**, Vale do Javari IT; **12**, Manu NP, Chandless SP, Cazumbá-Iracema ER; **13**, Madidi NP, Madidi NAIM, Apolobamba NAIM, Pilón Lajas BR, Bajo Madidi MPA, Tacana 1 IT, San José de Uchupiamonas IT, Pilón Lajas IT, Lecos de Apolo IT, Lecos de Larecaja IT; **14**, Noel Kempff Mercado NP.

^c^PCA categories are listed: BR, biosphere reserve; ER, extractive reserve; IT, indigenous territory; MPA, municipal protected area; NAIM, natural area under integrated management; NP, national park; NR, nature reserve; SDR, sustainable development reserve; SP, state park; WR; wildlife reserve.

^d^National entities providing financial and management support are listed: ARPA, Amazon Region Protected Areas Program; HECO, Herencia Colombia; PdP, Patrimonio Natural del Peru; MOS, Brazilian mosaicos under the Sistema Nacional de Unidades de Conservação.

CII, Contextual Intactness Index; GBMF, Gordon and Betty Moore Foundation; KLC, Key Landscape for Conservation; PCA, protected and conserved area.

As a proxy for management effectiveness and good governance, strongholds were identified particularly if countries had dedicated funding for management support of PCAs in a stronghold. In Brazil, that funding was provided by the Amazon Region Protected Areas Program (ARPA), which is coordinated by the Ministry of Environment and is the recipient of funds from multilateral and bilateral donors, international NGOs, and private foundations. In Colombia, a similar arrangement pertains to Herencia Colombia (HECO), which is managed by Parques Nacionales Naturales. In Peru, it is Patrimonio Natural del Peru (PdP), which receives institutional support through the National Protected Areas Service and financial support from external donors.

In Brazil, PCAs are already often grouped into larger management units, or “mosaicos,” so we considered these as strongholds. The intent of mosaicos is to operate at a larger scale and coordinate the management of government protected areas, neighboring indigenous territories and protected area buffer zones. Within the Brazilian Amazon, 4 large mosaicos were included in the list of strongholds: 1 (Eastern Amazonia) included mosaico da Amazônia Oriental; 3 (Apui–Southern Amazon) included two mosaicos, Apui and the mosaico da Amazônia Meridional; and 6 (Mamiráua–Amanã–Jaú–Unini) included mosaico Baixo Rio Negro.

**Fig 3:**
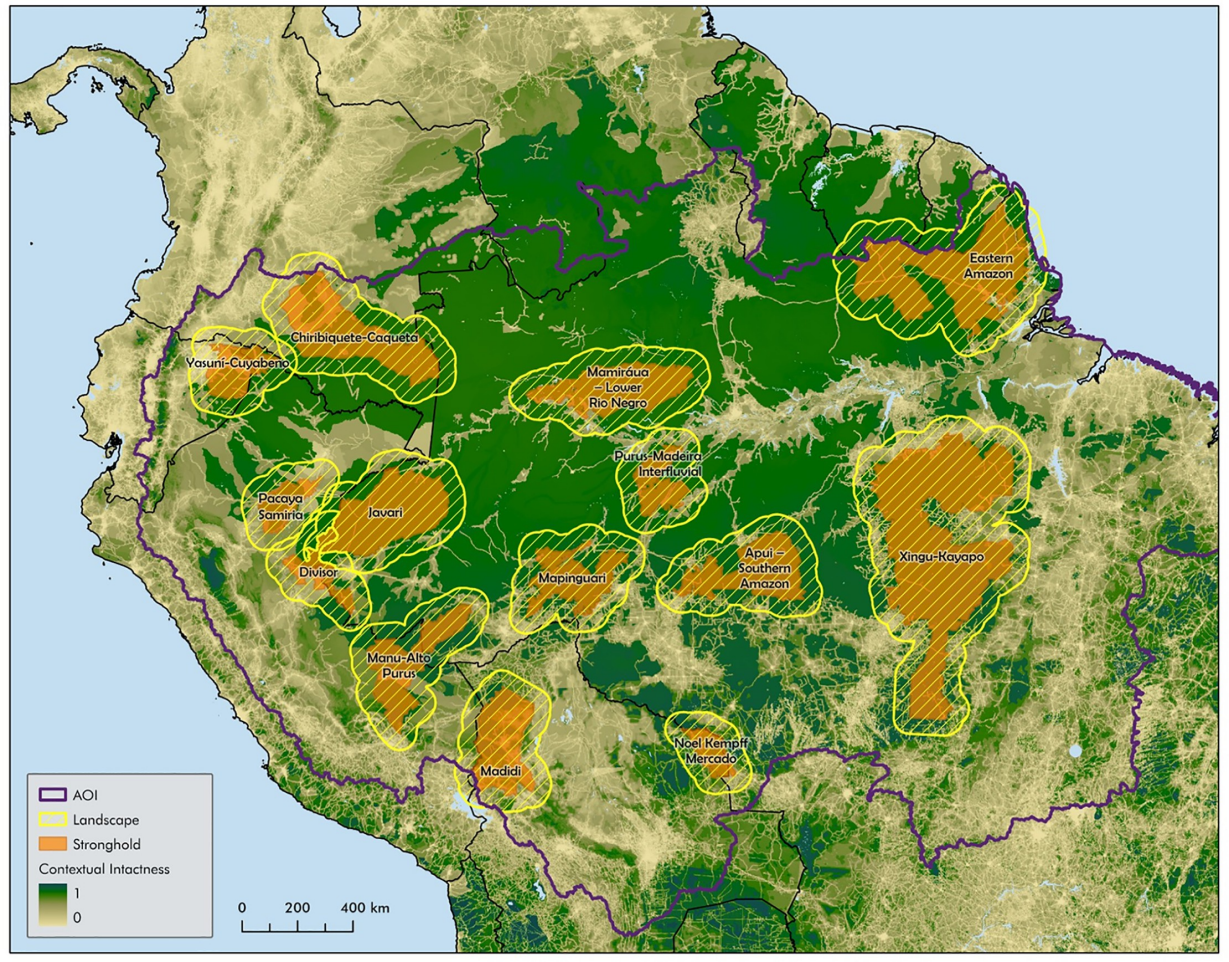
Nature’s Strongholds in Amazonia. Nature’s Strongholds embedded within conservation landscapes in Amazonia, mapped onto ecological integrity of the region. Data layers used listed in S2 Text. AOI, area of interest.

Fig 3 illustrates the 14 identified strongholds (average size = 69,808 km^2^) mapped onto the geographic distribution of ecological integrity across the basin. However, unlike in Central Africa, we did not use the boundaries of the KLCs [81] to define the conservation landscape. As in Central Africa, our intent was to compare the ecological integrity of strongholds to the matrix in which they were embedded, but the KLCs defined in Amazonia were very large and together covered much of the Amazon basin. Accordingly, Fig 3 plots the boundaries of each conservation landscape as an arbitrarily defined 60 km buffer around that stronghold (see S2 Text). In all cases, CII values of strongholds were greater than that of the surrounding landscape.

**Fig 4:**
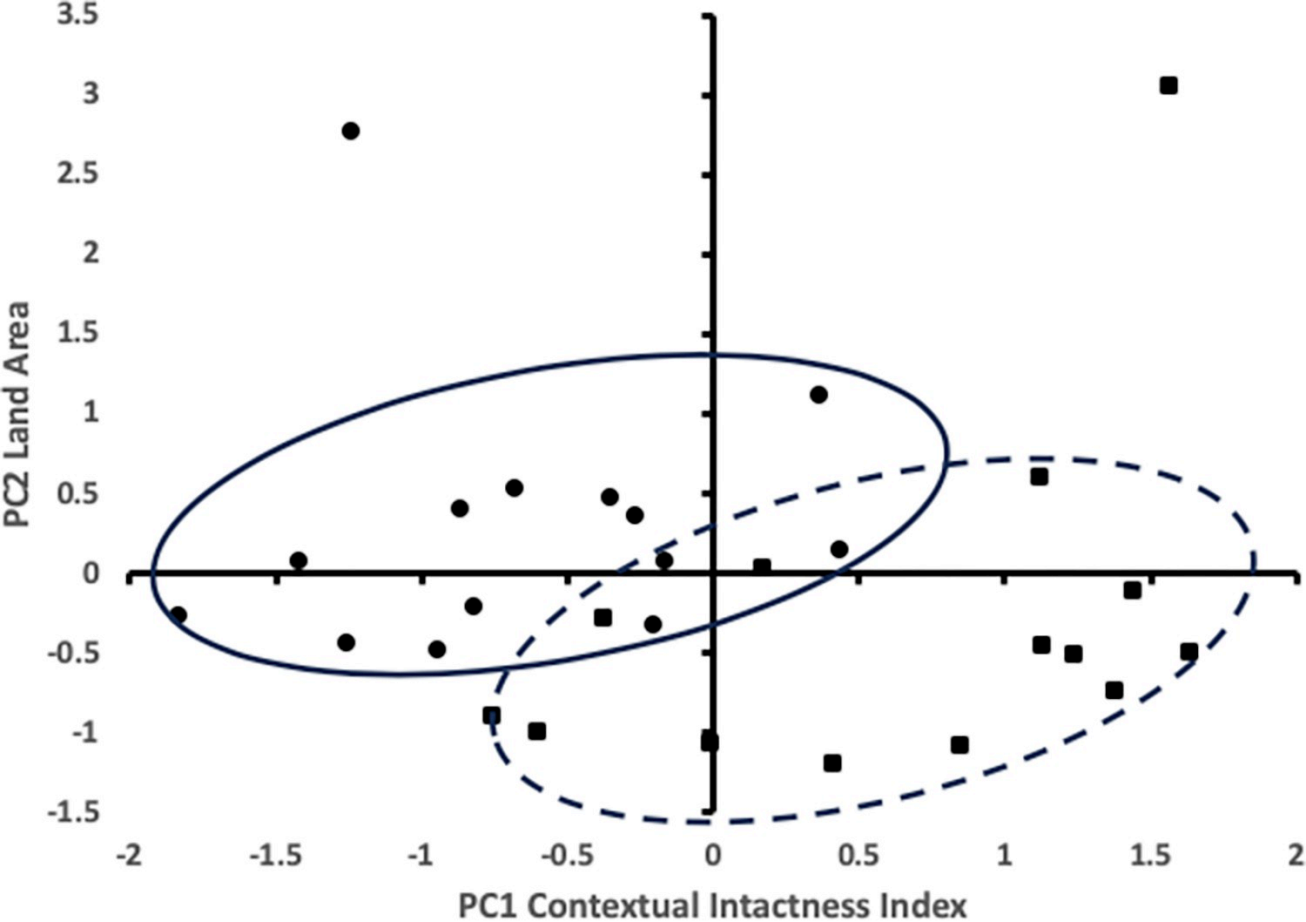
Ecological integrity of Nature’s Strongholds in Amazonia. Principal component analysis for Strongholds (filled squares, dashed oval) and surrounding conservation landscapes (filled circles, solid oval). Principal component 1 (PC1), which is most heavily weighted towards the CII, is plotted against principal component 2 (PC2), which is most heavily weighted towards land area. CII, Contextual Intactness Index.

To make the case that strongholds are more ecologically intact than the surrounding landscapes, we used principal component analysis to compare, for all 1 km grid cells, the CII, the standard deviation of the CII, and the land areas with values scaled to have a mean of 0 and a variance of 1 (see S2 Text and S4 Table). As in the Central Africa case study, principal component 1 is a function of high intactness (CII = 0.956, SD (CII) = −0.953, land area = 0.08), and principal component 2 is almost completely dominated by the size of the land area (land area = 0.996, SD (CII) = 0.087, CII = 0.003). The ovals in Fig 4 highlight the distribution of points in the stronghold class and the surrounding landscape class. They illustrate the degree of separation (not a statistical representation), the tendency for strongholds to be more ecologically intact than the surrounding landscape, and for larger strongholds to be more ecologically intact than smaller strongholds. These observations were confirmed using paired *t* tests, which indicated that strongholds have both a higher average ecological integrity and a lower variance in ecological integrity than the surrounding matrix (see S2 Text). We also confirmed that conservation landscapes that contained identified strongholds are more ecologically intact than the Amazon basin as a whole. The idea that Nature’s Strongholds and conservation landscapes are important for biodiversity conservation in the Amazon basin is suggested by strongholds having a higher ecological integrity, which infers a higher biodiversity value than the matrices in which they are embedded, and conservation landscapes (strongholds and surrounding matrices) have a higher ecological integrity than the Amazon basin as a whole (see S2 Text and S5 Table).

Another proxy for biodiversity and its distribution relative to strongholds and conservation landscapes is provided by Wallace and colleagues [82]. Although these authors did not focus on strongholds per se, they examined the geographic distribution of amphibian, mammal, and bird diversity in the GBMF conservation landscapes (termed “mosaics,” as distinct from Brazilian mosaicos). Documenting species occurrence in the Amazon remains incomplete, but the authors, based on the geo-referenced occurrences of species across the region, concluded that mosaics were disproportionately important for conserving biodiversity: “the 12 conservation mosaics cover 53.84% of the Amazon basin [but] are expected to hold 3,836 species, representing 66.64% of Amazon species.”

## Planning at the scale of Nature’s Strongholds

NGOs are increasingly organizing their area-based conservation efforts at the scale and complexity of strongholds. For example, the WCS and the World Wide Fund for Nature have structured their terrestrial area-based work around the conservation of large landscapes, which typically extend beyond parks and protected areas, encompassing a diverse range of land use categories [78]. This approach emphasizes the ecological integrity of these areas, that their conservation has consequences for nature and people, and that these large areas serve to protect and maintain a number of values, including biodiversity, watersheds, carbon stocks and sinks, traditional cultures, and human livelihoods.

Another example of planning at the scale and complexity of strongholds is provided by the German “Legacy Landscapes Fund” [79]. Legacy Landscapes are terrestrial areas across the globe that are larger than 2,000 km^2^, are ecologically intact, and are home to globally important biodiversity. Selected Legacy Landscapes receive significant long-term financing (1 million dollars a year for a minimum of 15 to 30 years). The Legacy Landscapes program explicitly recognizes the need to provide this financing at large, spatial scales. Each Legacy Landscape comprises a core protected area that covers at least 1,000 km^2^ (or at least 50% of the entire landscape), is IUCN Category I/II or equivalent, along with contiguous land categories such as community managed conservation areas, and/or other contiguous protected areas with a different IUCN status.

Range-wide priority setting for species-specific conservation efforts typically plan at this scale. A global conservation plan for jaguars identified 51 “jaguar conservation units” (JCUs), large, spatially defined areas with viable populations, often in and around protected areas. JCUs averaged over 25,000 km^2^ in size [90]. In a different socioeconomic and ecological context, Walston and colleagues [91] identified 42 “source sites” across the present range of tigers. Source sites were largely protected areas and defined as having the potential to maintain >25 breeding females, with the area having a conservation infrastructure and the legal mandate for protection. Their average size was 2,100 km^2^. Source sites offered the potential for tigers to expand across the wider landscape.

## Managing Nature’s Strongholds

Conservation management of potentially multiple categories of PCAs across large areas will require the integration of conservation planning systems and the administrative and participatory mechanisms to coordinate management. Some of the challenges to doing this are at least partly technical, such as ensuring that key indicators of management effectiveness are defined appropriately and measured at the appropriate scale [69]. Other challenges include the need to involve a wide range of stakeholders and secure approval for conservation action. To do so will require the engagement of carefully nested stakeholder groups, operating within individual jurisdictions and across the Nature’s Stronghold as a whole. Preventing takeover by a few powerful interest groups will require constant oversight and ensuring efficient and equitable engagement over a wide area will be challenging. Similarly, planning will need to take place at a wider level than hitherto, including with respect to current and future climate change. None of this is revolutionary, the tools and methods exist, but they have seldom been applied at this scale [92].

These are the challenges faced by the “Mosaicos de unidades de conservação” within the Brazilian national system of protected areas, which provide a pertinent example of how to approach the scaled-up management of multiple PCAs, often comprising different land categories. Mosaicos are spatially organized collections of different land use categories under different jurisdictions (see Case Study 2). When the Sistema Nacional de Unidades de Conservação was established, Article 26 of the 2000 Law 9.985 stated: “when there is a set of conservation units of different categories or not, close, juxtaposed or overlapping, and other public or private protected areas, constituting a mosaico, the management of the group should be carried out in an integrated and participatory manner, considering its different conservation objectives, in order to make the presence of biodiversity, the enhancement of socio-diversity and sustainable development in the regional context compatible.” The intent was to enable management integration across much larger areas and create economies of scale. For example, one of the oldest and most consolidated mosaicos in the Brazilian Amazon is the Lower Rio Negro mosaico, which includes 11 PCAs (national parks, sustainable development reserves, environmental protection areas, state parks, and extractive reserves) in 6 Amazonas municipalities covering an area of 74,128 km^2^ [93]. To date, conservation management has depended on strengthening systems within each PCA, but to improve management effectiveness across the whole mosaico, the governmental Instituto Chico Mendes de Conservação da Biodiversidade has recently established councils and management units (Núcleos de Gestão Integrada) for each.

## Extending the Nature’s Strongholds approach to other regions

Conserving at the scale of Nature’s Strongholds will support the efforts of governments and the conservation community to align and coordinate protection over the larger areas that are needed to address the global threats to biodiversity stemming from the loss and degradation of Nature. We have provided examples in Central Africa and Amazonia. Characteristics of strongholds will vary with the ecosystem, the size and spatial distribution of PCAs, the fragmentation or degree of continuity of natural habitats in the landscape, the spatial pattern of ecological integrity, and the existing governance and management regimes. The example of mosaicos in Brazil illustrates how the size of strongholds might vary. While the 4 mosaicos in the Brazilian Amazon are large (averaging 56,303 km^2^, with a median size of 50,876 km^2^), the 22 terrestrial mosaicos from other regions and ecosystems in Brazil average 8,960 km^2^, with a median size of 3,785 km^2^.

Identifying Nature’s Strongholds remains a work in progress, and also depends on advances in land-use planning, restoration of degraded sites, and the establishment of equitable and effective conservation management. The approach is clearly most suited to areas that contain large, connected areas of natural habitat with existing areas under conservation management, and is least applicable in scattered, fragmented ecosystems with a variety of different land uses. There are, for example, areas of Europe and Central Asia where large semi-natural areas still exist, such as the Carpathian and Caucasus mountains, and large, sparsely populated areas of Kazakhstan, Kyrgyzstan, and Tajikistan [94,95]. A combination of strategic land purchase and negotiated agreements with governments and other landowners could create conservation areas at scale. The expansion of deer, wolf, jackal, and lynx throughout Eastern Europe and Central Asia shows the potential for large-scale wildlife conservation in these areas [96].

## Conclusions

In this Essay, we argue that to meet the GBF’s 30x30 target, conservation areas need to be large enough to encompass functioning ecosystems and their associated biodiversity, and located in areas of high ecological integrity. Often, this will require well-connected aggregations of effectively managed and equitably governed PCAs, embedded in a larger conservation landscape. We have provided a framework to identify these areas, which we call Nature’s Strongholds, which have the characteristics of large size, interconnected PCAs, high ecological integrity, and effective management and good governance.

We interpret these characteristics in the context of Central Africa and Amazonia and identify strongholds within these 2 regions. Other strongholds might be included in the future if PCAs meet criteria of ecological integrity, adequate funding, management effectiveness, and good governance. When applying the approach to other regions, the specific characteristics used to identify strongholds will vary with the size and spatial distribution of PCAs, the matrix in which they are embedded (including patterns of ecological integrity), and management and governance regimes.

Governmental, non-governmental, and civil society organizations engaged with area-based conservation are increasingly planning and coordinating across large areas, multiple jurisdictions, and a diversity of management authorities. Management at this scale and degree of complexity, while challenging, will allow authorities to effectively contribute to biodiversity conservation and promote adaptation to climate change.

## Supporting information

S1 Table Size, mean, and standard deviation of Contextual Intactness Index (CII) for Key Landscapes for Conservation (KLCs) (excluding PCAs in identified strongholds) and PCAs in Central Africa. (DOCX)

S2 Table Size, mean, and standard deviation of Contextual Intactness Index (CII) for Key Landscapes for Conservation (KLCs) (including all PCAs in identified strongholds) in Central Africa. (DOCX)

S3 Table Present distribution and abundances of forest elephants and great apes across Nature’s Strongholds in Central Africa. (DOCX)

S4 Table Size, mean, and standard deviation of Contextual Intactness Index (CII) for Amazonian strongholds and the surrounding landscapes (considered separately). (DOCX)

S5 Table Size, mean, and standard deviation of Contextual Intactness Index (CII) for Amazonian strongholds and the surrounding landscapes (considered together). (DOCX)

S1 Text Identifying Key Conservation Landscapes and Nature’s Strongholds in Central Africa. (DOCX)

S2 Text Identifying Conservation Landscapes and Nature’s Strongholds in Amazonia. (DOCX)

## Abbreviations

CBD: Convention on Biological Diversity
CII: Contextual Intactness Index
EU: European Union
GBF: Global Biodiversity Framework
GBMF: Gordon and Betty Moore Foundation
ITT: indigenous and traditional territory
IUCN: International Union for the Conservation of Nature
JCU: jaguar conservation unit
KLC: Key Landscapes for Conservation
METT: Management Effectiveness Tracking Tool
NGO: non-governmental organization
OECM: other effective area-based conservation measure
PCA: protected and conserved area
WCS: Wildlife Conservation Society
