# Supplementary material for: Scaling up area-based conservation to implement the Global Biodiversity Framework’s 30x30 target: The role of Nature’s Strongholds
Source: PLoS Biol. 2024 May 21;22(5):e3002613. doi: 10.1371/journal.pbio.3002613 (PMC11108224; doi:10.1371/journal.pbio.3002613)
Supplement: S1 Table — (DOCX) [file pbio.3002613.s001.docx]

**Supplementary Table S1. Size, mean and standard deviation of Contextual Intactness Index (CII) for Key Landscapes for Conservation (KLCs) (excluding PCAs in identified strongholds) and PCAs in Central Africa.**

John G. Robinson^1*^ and Danielle LaBruna ^1^

1 Wildlife Conservation Society, Bronx, New York, USA.

^*^Corresponding author, email: [wildcons@gmail.com](mailto:wildcons@gmail.com)

| Numbers  follow  [1] | **Key Landscape for Conservation (KLC)** | **KLC**  **Count**  **N^[[1]](#endnote-1)^** | **KLC**  **Mean Contextual Intactness Index (CII)** | **KLC**  **Standard**  **Deviation** | **Stronghold**  (numbered sequentially, and listing individual PCAs) | **PCA**  **Count**  **N** | **PCA**  **Mean Contextual Intactness Index (CII)** | **PCA Standard**  **Deviation** |
| --- | --- | --- | --- | --- | --- | --- | --- | --- |
| CAF01 | Cross River – Takamanda – Mt. Cameroon - Korup | 25,933 | 0.3810 | 0.2143 | (1)  Cross River  Takamanda  Mt. Cameroun | 3,871  744  686 | 0.5376  0.6139  0.3160 | 0.0877  0.1128  0.0823 |
| CAF03 | Greater Tri-National  3a. Cameroon | 57,605 | 0.6875 | 0.2029 | (2)  Dja | 6,168 | 0.8154 | 0.0623 |
|  | 3b. Gabon | 70,802 | 0.7487 | 0.2559 | (3)  Lopé  Ivindo  Minkebe | 5,786  3,473  8,802 | 0.7522  0.8214  0.9131 | 0.2506  0.2027  0.1030 |
|  | 3c. Congo Republic | 41,031 | 0.8250 | 0.2107 | (4)  Odzala Kokoua | 15,928 | 0.8836 | 0.1322 |
|  | 3d. Sangha Tri-National (Cameroon, Congo Republic and CAR) | 68,689 | 0.7884 | 0.2064 | (5)  Dzanga - Sangha  Nouabalé-Ndoki  Lobéké  Lac Télé  Ntokou - Pikounda | 3,986  4,791  2,518  5,256  4,980 | 0.8210  0.9169  0.8061  0.8687  0.8389 | 0.1523  0.0309  0.1559  0.0626  0.2130 |
| CAF04 | Gamba-Mayumba-Conkouati | 49,744 | 0.4932 | 0.3008 | (6)  Gamba complex  (Loango, Iguela, Moukalaba doudou)  Conkouati | 13,655  4,612 | 0.7626  0.6981 | 0.2473  0.1945 |
| CAF05 | Garamaba-Bili Uere – Chinko – Zemongo – Southern  5a. CAR | 92,597 | 0.9100 | 0.1551 | (7)  Chinko | 29,015 | 0.9709 | 0.0624 |
| CAF05 | 5b. Dem. Rep. Congo | 111,074 | 0.6919 | 0.2312 | (8)  Garamba | 5,799 | 0.7171 | 0.1884 |
| CAF05 | 5c. South Sudan | 89,798 | 0.6016 | 0.1835 | (9)  Southern | 22,603 | 0.5916 | 0.1662 |
| CAF06 | Gounda- St. Floris – Bamingui and surrounding hunting blocks | 108,802 | 0.8970 | 0.1411 | (10)  Manovo – Gounda – St. Floris  Bamingui-Bangoran | 23,765  13,213 | 0.8971  0.9482 | 0.1377  0.0732 |
| CAF07 | Salonga | 38,928 | 0.7531 | 0.1985 | (11)  Salonga | 39,067 | 0.8148 | 0.1447 |
| CAF08 | Okapi | 27,661 | 0.6977 | 0.1888 | (12)  Okapi | 16,339 | 0.7044 | 0.1575 |
| CAF09 | Kahuzi-Biega | 13,435 | 0.5423 | 0.2740 | (13)  Kahuzi-Biega | 7,851 | 0.5696 | 0.2019 |
| CAF10 | Maiko-Tayna | 22,390 | 0.5762 | 0.2026 | (14)  Maiko | 12,826 | 0.7832 | 0.1480 |
| CAF14 | Itombwe-Kabobo  14a. Itombwe  14b. Kabobo | 5,165  5,730 | 0.2928  0.4570 | 0.2098  0.1527 | (15)  Itombwe  Kabobo | 7,070  2,177 | 0.4370  0.5295 | 0.1846  0.1343 |
| CAF15 | Lomami | 25,840 | 0.7737 | 0.1464 | (16)  Lomami | 10,358 | 0.7963 | 0.0920 |
| CAF16 | Mbam and Djerem | 14,695 | 0.6643 | 0.2105 | (17)  Mbam and Djerem  Deng Deng | 5,038  804 | 0.7825  0.6388 | 0.1379  0.1384 |
| CAF18 | Zakouma – Sinlaka Minla | 22,771 | 0.5785 | 0.1842 | (18)  Zakouma  Siniaka – Minla  Bahr Salamat | 3,628  5,111  25,079 | 0.6288  0.7528  0.5933 | 0.1840  0.1468  0.1882 |

1. The discrepancy between the actual areas of Nature’s Strongholds and KLCs (from Table 1) and the Counts (N) of the Contextual Intactness Index arises because the CII relies on the CSIRO (Commonwealth Scientific and Industrial Research Organization) use of the BILBI dataset (Biogeographic modelling Infrastructure for Large-scaled Biodiversity Indicators). BILBI has grid cells of 30-arcseconds or only approximately 1km.

   **References**

   European Union. Larger than Elephants: Inputs for an EU strategic approach to wildlife conservation in Africa, 2015 Brussels, Belgium: European Commission, Directorate-General for International Cooperation and Development. ISBN 978-92-79-49564-9, doi:10.2841/909032. [↑](#endnote-ref-1)
