# Supplementary material for: Scaling up area-based conservation to implement the Global Biodiversity Framework’s 30x30 target: The role of Nature’s Strongholds
Source: PLoS Biol. 2024 May 21;22(5):e3002613. doi: 10.1371/journal.pbio.3002613 (PMC11108224; doi:10.1371/journal.pbio.3002613)
Supplement: S2 Table — (DOCX) [file pbio.3002613.s002.docx]

**Supplementary Table S2. Size, mean and standard deviation of Contextual Intactness Index (CII) for Key Landscapes for Conservation (KLCs) (including all PCAs in identified strongholds) in Central Africa**

John G. Robinson^1*^ and Danielle LaBruna ^1^

1 Wildlife Conservation Society, Bronx, New York, USA.

^*^Corresponding author, email: [wildcons@gmail.com](mailto:wildcons@gmail.com)

| Numbers follow  [1] | **Key Landscape for Conservation (KLC)** | **KLC**  **Count**  **N** | **KLC**  **Mean Contextual Intactness Index (CII)** | **KLC**  **Standard**  **Deviation** |
| --- | --- | --- | --- | --- |
| CAF01 | Cross River – Takamanda – Mt. Cameroon - Korup | 31,207 | 0.4044 | 0.2084 |
| CAF03 | Greater Tri-National  3a. Cameroon | 63,783 | 0.6999 | 0.1975 |
|  | 3b. Gabon | 89,106 | 0.7685 | 0.2477 |
|  | 3c. Congo Republic | 56,706 | 0.8409 | 0.1941 |
|  | 3d. Sangha Tri-National (Cameroon, Congo Republic and CAR) | 90,220 | 0.8046 | 0.1952 |
| CAF04 | Gamba-Mayumba-Conkouati | 68,011 | 0.5612 | 0.3063 |

| CAF05 | Garamaba-Bili Uere – Chinko – Zemongo – Southern  5a. CAR | 121,612 | 0.9207 | 0.1416 |
| --- | --- | --- | --- | --- |
|  | 5b. Dem. Rep. Congo | 116,871 | 0.6932 | 0.2293 |
|  | 5c. South Sudan | 112,403 | 0.5996 | 0.1802 |
| CAF06 | Gounda- St. Floris – Bamingui and surrounding hunting blocks | 145,666 | 0.9016 | 0.1366 |
| CAF07 | Salonga | 77,995 | 0.7840 | 0.1764 |
| CAF08 | Okapi | 44,000 | 0.7002 | 0.1779 |
| CAF09 | Kahuzi-Biega | 21,286 | 0.5524 | 0.2502 |
| CAF10 | Maiko-Tayna | 35,216 | 0.6516 | 0.2097 |
| CAF14 | Itombwe-Kabobo  14a. Itombwe  14b. Kabobo | 12,235  7,907 | 0.3761  0.4769 | 0.2082  0.1513 |
| CAF15 | Lomami | 36,198 | 0.7802 | 0.1335 |
| CAF16 | Mbam and Djerem | 20,537 | 0.6923 | 0.1995 |
| CAF18 | Zakouma – Sinlaka Minla | 56,589 | 0.6041 | 0.1893 |
|  |  |  |  |  |
| Congo  Basin | (excluding the landscapes) | 3,839,210 | 0.5694 | 0.2447 |

**References**

1. European Union. Larger than Elephants: Inputs for an EU strategic approach to wildlife conservation in Africa, 2015 Brussels, Belgium: European Commission, Directorate-General for International Cooperation and Development. ISBN 978-92-79-49564-9, doi:10.2841/909032.
