# Supplementary material for: Scaling up area-based conservation to implement the Global Biodiversity Framework’s 30x30 target: The role of Nature’s Strongholds
Source: PLoS Biol. 2024 May 21;22(5):e3002613. doi: 10.1371/journal.pbio.3002613 (PMC11108224; doi:10.1371/journal.pbio.3002613)
Supplement: S3 Table — (DOCX) [file pbio.3002613.s003.docx]

**Supplementary Table S3. Present Distribution and Abundances of Forest Elephants and Great Apes across Nature’s Strongholds in Central Africa.**

John G. Robinson^1*^ and Fiona Maisels^2^

1 Wildlife Conservation Society, Bronx, New York, USA.

^2^ WCS Congo, Brazzaville, Republic of Congo, and Biological and Environmental Sciences,

University of Stirling, UK>

^*^Corresponding author, email: [wildcons@gmail.com](mailto:wildcons@gmail.com)

| Key Landscape for Conservation (KLC) | Stronghold (individual PCAs listed) | Forest Elephants | Cross River  Gorilla | Grauer’s  Gorilla | Western Lowland Gorilla | Central  Chimpanzee | Nigeria-Cameroon Chimpanzee | Eastern Chimpanzee | Bonobo |
| --- | --- | --- | --- | --- | --- | --- | --- | --- | --- |
| Cross River – Takamanda – Mt. Cameroon - Korup | Cross River  Takamanda  Mt. Cameroon  (1) | X  present  X | X  X |  |  |  | XX  present |  |  |
| Greater Tri-National  3a. Cameroon | Dja  (2) | XX |  |  | XXX | XXX |  |  |  |
| 3b. Gabon | Lopé  Ivindo  Minkebe  (3) | XXX  XXX  XXX |  |  | XXX  XXX  present | XXX  XXX  present |  |  |  |
| 3c. Republic of Congo | Odzala - Kokoua  (4) | XXX |  |  | XXXX | XXX |  |  |  |
| 3d. Sangha Tri-National (Cameroon, Republic of Congo and CAR) | Dzanga - Sangha  Nouabalé - Ndoki  Lobéké  Lac Télé  Ntokou –  Pikounda  (5) | XX  XXX  XX  XX  XXX |  |  | XXX  XXX  XXX  XXX  XXX | XX  XXX  XX  XX  XXX |  |  |  |
| Gamba-Mayumba-Conkouati | Gamba complex  (Loango,  Moukalaba-  Doudou)  Conkouati  (6) | XXX |  |  | XX  XXX | XX  XX |  |  |  |
| Garamba-Bili Uere – Chinko – Zemongo – Southern  5a. CAR | Chinko  (7) | X |  |  |  |  |  | XX |  |
| 5b. Dem. Rep. Congo | Garamba  (8) | XXX |  |  |  |  |  | XX |  |
| 5c. South Sudan | Southern  (9) |  |  |  |  |  |  |  |  |
| Gounda- St. Floris – Bamingui and surrounding hunting blocks | Manovo – Gounda – St. Floris  Bamingui-Bangoran  (10) | present |  |  |  |  |  |  |  |
| Salonga | Salonga  (11) | XXX |  |  |  |  |  |  | XXXX |
| Okapi | Okapi  (12) | XX |  |  |  |  |  | XXXX |  |
| Kahuzi-Biega | Kahuzi-Biega  (13) | X |  | XXX |  |  |  | XX |  |
| Maiko-Tayna | Maiko  (14) | X |  | XX |  |  |  | X |  |
| Itombwe-Kabobo  14a.  14b. | Itombwe  Kabobo  (15) | present |  | X |  |  |  | XXX  XXX |  |
| Lomami | Lomami  (16) | XX |  |  |  |  |  |  | XXXX |
| Mbam and Djerem | Mbam Djerem  Deng Deng  (17) | XX |  |  | XX | XX | XX |  |  |
| Zakouma – Sinlaka Minla | Zakouma  Siniaka – Minla  Bahr Salamat  (18) | XX |  |  |  |  |  |  |  |

XXXX = Population in the tens of thousands, XXX = Population in the thousands, XX = Population in the hundreds, X = Population in the tens, “present” indicates recent records. Population abundance estimates for Elephants from Thouless et al. [1], and Great Apes from Rainer et al. [2]. Shaded cells indicate areas outside the historical geographic range of individual species.

References

1. Thouless CR, Dublin HT, Blanc JJ, Skinner DP, Daniel TE, Maisels F, et al. African Elephant Status Report 2016: an update from the African Elephant database. Gland, Switzerland: Occasional Paper Series of the IUCN Species Survival Commission, No. 60 IUCN/SSC African Elephant Specialist Group; 2016
2. Rainer H, White A, Lanjouw A, editors. State of the Apes: Killing, capture, trade and conservation. Cambridge: Cambridge University Press; 2021
