# Supplementary material for: Scaling up area-based conservation to implement the Global Biodiversity Framework’s 30x30 target: The role of Nature’s Strongholds
Source: PLoS Biol. 2024 May 21;22(5):e3002613. doi: 10.1371/journal.pbio.3002613 (PMC11108224; doi:10.1371/journal.pbio.3002613)
Supplement: S4 Table — (DOCX) [file pbio.3002613.s004.docx]

**Supplementary Table S4. Size, mean and standard deviation of Contextual Intactness Index (CII) for Amazonian strongholds and the surrounding landscapes (considered separately)**

John G. Robinson^1*^ and Danielle LaBruna ^1^

1 Wildlife Conservation Society, Bronx, New York, USA.

^*^ email: wildcons@gmail.com

| **Stronghold** | **Stronghold**  **Count**  **N^[[1]](#endnote-1)^** | **Stronghold**  **Mean Contextual Intactness Index (CII)** | **Stronghold**  **Standard**  **Deviation** | **Surrounding Landscape Count**  **N** | **Surrounding Landscape**  **Mean Contextual Intactness Index (CII)** | **Surrounding**  **Landscape**  **Standard**  **Deviation** |
| --- | --- | --- | --- | --- | --- | --- |
| (1)  Eastern Amazon | 145,503 | 0.7426 | 0.0996 | 173,062 | 0.6989 | 0.1589 |
| (2)  Xingu - Kayapo | 310,145 | 0.7932 | 0.0923 | 276,038 | 0.5261 | 0.2407 |
| (3)  Apui –  Southern Amazon | 74,356 | 0.7948 | 0.0689 | 117,472 | 0.6768 | 0.2110 |
| (4)  Purus – Madeira Interfluvial  (5)  Mapinguari | 30,983  56,599 | 0.7238  0.7823 | 0.1104  0.0880 | 74,907  118,906 | 0.6439  0.6006 | 0.1822  0.2319 |
| (6)  Mamiráua – Amanã – Jaú | 76,817 | 0.7166 | 0.0787 | 110,230 | 0.6874 | 0.1403 |
| (7)  Yasuní-Cuyabeno | 34,004 | 0.5935 | 0.2098 | 71,556 | 0.4689 | 0.2575 |
| (8)  Chiribiquete – Caqueta | 101,405 | 0.6591 | 0.1515 | 129,417 | 0.5897 | 0.2059 |
| (9)  Pacaya Samiria | 25,346 | 0.6744 | 0.1747 | 64,911 | 0.4822 | 0.2019 |
| (10)  Divisor | 26,893 | 0.6270 | 0.2117 | 79,420 | 0.5785 | 0.2111 |
| (11)  Javari | 100,346 | 0.7527 | 0.0669 | 101,029 | 0.6533 | 0.1848 |
| (12)  Manu –  Alto Purús | 69,199 | 0.7881 | 0.1080 | 126,327 | 0.6463 | 0.2034 |
| (13)  Madidi | 76,290 | 0.6316 | 0.1936 | 101,020 | 0.4297 | 0.1919 |
| (14)  Noel Kempff Mercado | 22,364 | 0.6591 | 0.1195 | 60,246 | 0.5703 | 0.2192 |

1. The discrepancy between the actual areas of Nature’s Strongholds and Surrounding Landscapes (from Table 2) and the Counts (N) of the Contextual Intactness Index arises because the CII relies on the CSIRO (Commonwealth Scientific and Industrial Research Organization) use of the BILBI dataset (Biogeographic modelling Infrastructure for Large-scaled Biodiversity Indicators). BILBI has grid cells of 30-arcseconds or only approximately 1km. [↑](#endnote-ref-1)
