# Supplementary material for: Scaling up area-based conservation to implement the Global Biodiversity Framework’s 30x30 target: The role of Nature’s Strongholds
Source: PLoS Biol. 2024 May 21;22(5):e3002613. doi: 10.1371/journal.pbio.3002613 (PMC11108224; doi:10.1371/journal.pbio.3002613)
Supplement: S5 Table — (DOCX) [file pbio.3002613.s005.docx]

**Supplementary Table S5. Size, mean and standard deviation of Contextual Intactness Index (CII) for Amazonian strongholds and the surrounding landscapes (considered together)**

John G. Robinson^1*^ and Danielle LaBruna ^1^

1 Wildlife Conservation Society, Bronx, New York, USA.

^*^Corresponding author, email: [wildcons@gmail.com](mailto:wildcons@gmail.com)

| **Stronghold** | **Stronghold & Surrounding**  **Landscape**  **Count**  **N** | | **Stronghold & Surrounding Landscape**  **Mean Contextual Intactness Index** | **Stronghold & Surrounding Landscape**  **Standard**  **Deviation** |
| --- | --- | --- | --- | --- |
| Eastern Amazon | 318,565 | 0.7170 | | 0.1369 |
| Xingu - Kayapo | 586,183 | 0.6675 | | 0.2226 |
| Apui –  Southern Amazon | 191,828 | 0.7225 | | 0.1801 |
| Purus – Madeira Interfluvial  Mapinguari | 105,890  175,505 | 0.6673  0.6592 | | 0.1684  0.2144 |
| Mamiráua – Amanã – Jaú | 187,047 | 0.6993 | | 0.1198 |
| Yasuní-Cuyabeno | 105,560 | 0.5091 | | 0.2500 |
| Chiribiquete – Caqueta | 230,822 | 0.6202 | | 0.1872 |
| Pacaya Samiria | 90,257 | 0.5361 | | 0.2129 |
| Divisor | 106,313 | 0.5908 | | 0.2123 |
| Javari | 201,375 | 0.7028 | | 0.1477 |
| Manu –  Alto Purús | 195,526 | 0.6965 | | 0.1883 |
| Madidi | 177,310 | 0.5169 | | 0.2170 |
| Noel Kempff Mercado | 82,610 | 0.5944 | | 0.2012 |
| Amazon Basin (excluding landscapes) | 5,868,088 | 0.5305 | | 0.2356 |
