# Supplementary material for: Scaling up area-based conservation to implement the Global Biodiversity Framework’s 30x30 target: The role of Nature’s Strongholds
Source: PLoS Biol. 2024 May 21;22(5):e3002613. doi: 10.1371/journal.pbio.3002613 (PMC11108224; doi:10.1371/journal.pbio.3002613)
Supplement: S1 Text — (DOCX) [file pbio.3002613.s006.docx]

**Supplementary Text S1. Identifying Key Conservation Landscapes and Nature’s Strongholds in Central Africa.**

John G. Robinson^1*,^ Danielle LaBruna ^1^, Tim O’Brien ^2^, Peter J. Clyne ^1,^ Hedley Grantham^3, 4^, Margaret Kinnaird ^5^, Fiona Maisels ^6, 7^, and Emma Stokes ^1^.

1 Wildlife Conservation Society, Bronx, New York, USA. ^2^ 34 Kibo Lane, Karen, Kenya. ^3^ Center for Ecosystem Science, School of Biological, Earth and Environmental Sciences, University of New South Wales, Australia. ^4^ Bush Heritage Australia, Melbourne, Victoria, Australia. ^5^ WWF – International, Gland, Switzerland. ^6^ WCS Congo, Brazzaville, Republic of Congo. ^7^ Biological and Environmental Sciences, University of Stirling, UK.

^*^Corresponding author, email: [wildcons@gmail.com](mailto:wildcons@gmail.com)

The European Union [1] defined a set of criteria to identify the most important Key Landscapes for Conservation (KLCs) in Africa. Within Central Africa, 20 such landscapes were identified (CAF01 – CAF20, numbers followed here). The KLC polygons were recently proposed for modification [2] and we followed those proposals here. Not included in this analysis were CAF02, CAF11 which we considered as outside the Central Africa basin. Recognizing the different management partners and the management contexts in the different countries, we subdivided the two largest landscapes, CAF03 and CAF05, to reflect national boundaries. The exception was that we treated the Sangha Tri-National (Cameroon, Congo Republic and CAR), where management efforts are more coordinated, as a single conservation landscape. CAF14 is a discontinuous conservation landscape, and the two blocks were considered separately.

The area of each KCL was based on the boundaries defined in European Union study [1] as modified in subsequent revisions (C. Aveling, pers. Comm., 27 Jan 2022). Further minor boundary revisions were made to encompass boundaries of PCAs.

Nature’s Strongholds were identified in 13 KLCs. CAF12, 13, 17, 19, and 20 contained conserved and managed areas, when only considering effectively managed areas, but these were smaller than the criterion of 5000 km^2^. Boundaries of strongholds were defined by the boundaries of conserved and managed areas (which included both protected areas and other conservation areas) from UNEP-WCMC and IUCN [3].

To describe the ecological integrity of KLCs and strongholds, we used Mokany et al. [4,5] ‘Contextual Intactness Index’ (CII) of all 1-km grid cells for all strongholds and for the surrounding KLC landscape. The index uses the Human Footprint Index [6], a measure of human pressure, and infers a biodiversity value based on geographically explicit species occurrence from museum collections. Mean CII values, standard deviations and cell counts for the 32 strongholds and for their 19 surrounding KLC polygons (sans the strongholds within) are presented in Supplementary Table S1. The CII index varies from 0 to 1, and calculates for each grid cell the proportion of cells which supported a similar assemblage of species but suffered a higher impact from human activities (based on the Human Footprint Index (HFP) [53]). High-value ecologically intact grid cells are those with a CII greater than 0.5. These are locations which are in better condition (lower HFP) than more than half of similar habitat.

Therefore, the following data layers used in Figure 1a:

- Contextual Intactness Index (CII) from Mokany et al. [5]. The index uses the Human Footprint Index [6], a measure of human pressure, and infers a biodiversity value based on geographically explicit species occurrence from museum collections. The index measures “the proportion of all those locations expected to have once supported a similar assemblage of species to the focal grid cell, but which have suffered higher impact from human activities than that cell” [4].
- Key Landscapes for Conservation (KLCs) from Weynant et al. [2].
- Stronghold layers adapted from UNEP-WCMC and IUCN Protected Planet [3]
- AOI boundary (Central Africa river basin) followed political boundaries in the south and east, and ecological boundaries (forest ecosystems) in the north.
- Political boundaries from @EuroGeographics (European Union GIS unit) and UN-FAO.

To assess whether strongholds are more ecologically intact than the KLCs in which they are embedded (excluding the area of the stronghold itself), we used a principal component analysis. We used data standardization so that land area values were transformed so that values had a mean of 0 and a variance of 1. The first component (PC1) is most heavily weighted toward the Contextual Intactness Index itself (0.893), followed by decreasing standard deviation (- 0.654) and size of KLCs and strongholds (0.406). This means that with higher values of PC1, ecological integrity, and area increases, while the standard deviation decreases. PC2 is primarily weighted by size of the area (0.846), increasing standard deviation of the CII (0.650) and least by the index itself (0.091). Each axis accounts for similar levels of variance in the data: 46.3% for PC1 and 38.2% for PC2, for a total of 84.5% of the total variance.

Using a paired t-test separately on the x-axis and y-axis is now justified because the x and y axes are not correlated and the tests are independent. The paired t-test for the x-axis (ecological intactness) is significant (t = -2.319, df = 12, P<0.05). The test for the y-axis (area) is also significant (t = 2.744, df = 12, P<0.05). We conclude that although surrounding KLCs tend to be larger, strongholds have both a higher and more consistent ecological integrity, as measured by the mean and coefficient of variation of the CII.

To assess whether KLCs (including the embedded stronghold) were more ecologically intact than the Congo basin as a whole, comparisons again used the CII of all 1-km grid cells (see Supplementary Table S2) The mean CII value for KLCs (including the Stronghold(s) within) and for the Congo basin were calculated using the Zonal Statistics as table tool in ArcGIS Pro [7]. The boundaries of the Central Africa basin were delineated as the approximate forest ecotone in the north, the ocean to the west, the mountains of the Great Rift Valley to the east, and the national political boundaries in the south. CAF18 falls outside this definition of the Central Africa basin, as does a small portion of CAF6. The combined mean Contextual Intactness of all 1-km grid cells of all KLCs (including the embedded stronghold) was 0.6481 (SD = 0.1704), which was greater (1-sided t-test = 1.666, df = 12, 0.05 < P < 0.1, mean difference = 0.07) than the CII mean (0.5694, SD = 0.2447) of the Congo Basin as a whole (excluding the land within the KLCs).

**References**

1. European Union. Larger than Elephants: Inputs for an EU strategic approach to wildlife conservation in Africa, 2015 Brussels, Belgium: European Commission, Directorate-General for International Cooperation and Development. ISBN 978-92-79-49564-9, doi:10.2841/909032.
2. Weynants M, Aveling C, Olivier R, Murray M. Key landscapes for the conservation of biodiversity in Sub-Saharan Africa. Proposal for modification of polygons; 2020. BIOPAMA. (1.0.0). Available from: <https://doi.org/10.5281/zenodo.8207555>
3. United Nations Environmental Programme (UNEP): World Conservation Monitoring Centre (WCMC) and International Union for the Conservation of Nature (IUCN). Protected Planet: The World Database on Protected Areas (WDPA) [On-line], [cited 2022 Mar 3}. Available from: [www.protectedplanet.net](http://www.protectedplanet.net)
4. Mokany K, Ferrier S, Harwood TD, Ware C, Di Marco M, Grantham HS, et al. Reconciling global priorities for conserving biodiversity habitat. PNAS 2020;117: 9906-9911.
5. Mokany K, Ferrier S, Harwood T, Ware C, Di Marco M, Grantham H, et al. Contextual intactness of habitat for biodiversity: global extent, 30 arcsecond resolution. v1. CSIRO. Data Collection. Licensed under [CC BY 4.0](https://creativecommons.org/licenses/by/4.0/) [doi: 10.25919/5e7854cfcb97e](https://doi.org/10.25919/5e7854cfcb97e).
6. Sanderson EW, Jaiteh M, Levy MA, Redford KH, Wannebo AV, Woolmer G. The Human Footprint and the Last of the Wild. BioScience 2002;52: 891-904.
7. Environmental Systems Research Institute (ESRI). ArcGIS Pro (Version 3.1.2). Available from: https://www.esri.com/en-us/arcgis/products/arcgis-pro/overview
