# Supplementary material for: Scaling up area-based conservation to implement the Global Biodiversity Framework’s 30x30 target: The role of Nature’s Strongholds
Source: PLoS Biol. 2024 May 21;22(5):e3002613. doi: 10.1371/journal.pbio.3002613 (PMC11108224; doi:10.1371/journal.pbio.3002613)
Supplement: S2 Text — (DOCX) [file pbio.3002613.s007.docx]

**Supplementary Text S2. Identifying Conservation Landscapes and Nature’s Strongholds in Amazonia.**

John G. Robinson^1*,^ Danielle LaBruna ^1^, Tim O’Brien ^2^, Peter J. Clyne ^1,^, Avecita Chicchon ^3^, Carlos Durigan ^4^, Adriana Moreira ^5^

1 Wildlife Conservation Society, Bronx, New York, USA. ^2^ 34 Kibo Lane, Karen, Kenya.

^3^ Andes-Amazon Initiative, Gordon and Betty Moore Foundation, Palo Alto, California, USA.

^4^ WCS Brasil, Manaus, Amazonas, Brazil. ^5^ Global Environmental Facility, Washington, D.C., USA.

A similar process was used to identify conservation landscapes and strongholds in Amazonia, although the specific methodology differed. Identification of strongholds depended in part on the expertise and opinions of the co-authors and other conservation colleagues. Boundaries of strongholds were defined by the boundaries of conserved and managed areas (which included both protected areas and other conservation areas) from UNEP-WCMC and IUCN [1].

The existence of conservation landscapes in Amazonia was informed by two recent efforts. First, the European Union [2] identified key landscapes for conservation (KLCs) in Latin America and the Caribbean. In the Amazon Basin, 17 such landscapes were identified (numbered 41-46, 50-60). Only those landscapes which included identified strongholds are listed here. Second, the Gordon and Betty Moore Foundation (GBMF), a major private funder for conservation in the Amazon basin, identified large landscapes (which they termed ‘mosaics’) [3]. We aligned these with KLCs. The EU analysis identified 17 landscapes in the Amazon basin, and the GBMF identified 12.

However, while KLCs or GBMF mosaics were helpful to delineate strongholds, both the areas of both KLCs and GBMF mosaics were very large, identified strongholds did not nest completely within KLCs, and some identified strongholds fell outside the KLCs. Accordingly, we first defined Nature’s Strongholds, and subsequently arbitrarily defined a matrix in which they were embedded as a 60 km buffer around each identified stronghold. 14 strongholds were identified. 13 of these were located within KLCs, and one was located outside of a KLC. All of the GBMF mosaics contain identified strongholds. 14 landscape matrices were delineated with a 60 km buffer surrounding the 14 Strongholds, except where that buffer would overlap with a nearby stronghold as this would compromise the mean CII values of the landscape matrix. For example, the Javari landscape was drawn so that it does not overlap neighboring Divisor stronghold. Strongholds are significantly smaller than the surrounding matrices with a mean ratio of stronghold size to the size of surrounding matrix of 0.632 (SD = 0.244, 95%, CI = [0.508 – 0.755]). Overlap of neighboring landscape polygons was allowed as this did not pose a compromise to landscape mean CII values.

Therefore, the following data layers used in Figure 1b:

- Contextual Intactness Index (CII) from Mokany et al. [4,5]. The index uses the Human Footprint Index [6], a measure of human pressure, and infers a biodiversity value based on geographically explicit species occurrence from museum collections.
- Landscape and Stronghold layers adapted from UNEP-WCMC and IUCN Protected Planet [1].
- AOI (Amazon river basin) is Basil Level 1 from [7]
- Political boundaries from @EuroGeographics (European Union GIS unit) and UN-FAO.

To compare CII values of Strongholds to their surrounding landscape matrices, mean CII values, standard deviations and cell counts were calculated for each of the 14 Strongholds and the 14 landscape matrices using the Zonal Statistics as Table tool in ArcGIS Pro [8] (see Supplementary Table 3). Comparisons used a principal component analysis. Data was standardized so that land area values were transformed so that values had a mean of zero and a variance of 1. To measure relative intactness, we used the CII (0 – 1) and the standard deviation of intactness. The resulting two variables, PC1 and PC2, account for 94% of the variation in the data. As in the Central Africa example, the first variable is a function of high intactness and low standard deviation (CII = 0.956, SD = -0.953, land area = 0.08). The second variable is almost completely dominated by size of the conservation area (Intactness index = 0.003, SD = 0.087, land area = 0.996). Strongholds consistently have a lower coefficient of variation than the surrounding matrix (Average stronghold CII = 0.71, CV = 0.19; surrounding matrix average CII = 0.59, CV = 0.36) indicating that ecological integrity values are less variable within the stronghold compared to the surrounding matrix. The CV for strongholds declines rapidly as ecological intactness increases compared to surrounding matrices as well, suggesting more consistent intactness in the most ecologically intact strongholds.

To assess whether conservation landscapes (both the stronghold and the surrounding matrix defined by the 60 km buffer) were more ecologically intact than the Amazon basin as a whole, comparisons again used the CII of all 1-km grid cells. Again, calculations of mean CII, standard deviations and cell counts were conducted using the Zonal Statistics as Table tool in ArcGIS Pro [8] (see Supplementary Table S4). The boundaries of Amazon Basin were defined by the Basin Level 1 polygon from the Amazon Aquatic Ecosystem Spatial Framework [7]. The combined mean Contextual Intactness of all 1-km grid cells of all conservation landscapes (both the stronghold and the surrounding matrix) was CII = 0.6541 (SD = 0.077), which was greater (1-sided t-test = 5.244, df = 13, P < 0.001, mean difference = 0.1503) than the CII mean (0.5305, SD = 0.2356) of the Amazon Basin as a whole (excluding the land within the conservation landscapes).

**References**

1. United Nations Environmental Programme (UNEP): World Conservation Monitoring Centre (WCMC) and International Union for the Conservation of Nature (IUCN). Protected Planet: The World Database on Protected Areas (WDPA) [On-line], [cited 2022 Mar 3}. Available from: [www.protectedplanet.net](http://www.protectedplanet.net)
2. European Union. Larger than Jaguars: Inputs for a strategic approach to biodiversity conservation in Latin America and the Caribbean. Luxembourg: Publications Office of the European Union. ISBN 978-92-76-17345-8, doi:10.2841/760354
3. Wallace R, Torrico O, Porcel Z, Domic E. Terrestrial vertebrate biological diversity in twelve Andes-Amazon conservation mosaics. La Paz, Bolivia: Gordon and Betty Moore Foundation and Wildlife Conservation Society; 2020.
4. Mokany K, Ferrier S, Harwood TD, Ware C, Di Marco M, Grantham HS, et al. Reconciling global priorities for conserving biodiversity habitat. PNAS 2020;117: 9906-9911.
5. Mokany K, Ferrier S, Harwood T, Ware C, Di Marco M, Grantham H, et al. Contextual intactness of habitat for biodiversity: global extent, 30 arcsecond resolution. v1. CSIRO. Data Collection. Licensed under [CC BY 4.0](https://creativecommons.org/licenses/by/4.0/) [doi: 10.25919/5e7854cfcb97e](https://doi.org/10.25919/5e7854cfcb97e).
6. Sanderson EW, Jaiteh M, Levy MA, Redford KH, Wannebo AV, Woolmer G. The Human Footprint and the Last of the Wild. BioScience 2002;52: 891-904.
7. Venticinque E, Forsberg B, Barthem RB, Petry P, Hass L, Mercado A, et al.  SNAPP Western Amazon Group - ﻿Amazon Aquatic Ecosystem Spatial Framework. Knowledge Network for Biocomplexity, 2016. Available from:  [doi:10.5063/F1BG2KX8](https://doi.org/10.5063/F1BG2KX8). Licensed under [CC BY 4.0](https://creativecommons.org/licenses/by/4.0/).
8. Environmental Systems Research Institute (ESRI). ArcGIS Pro (Version 3.1.2). Available from: https://www.esri.com/en-us/arcgis/products/arcgis-pro/overview
